# A randomized study of the effect of patient positioning on setup reproducibility and dose distribution to organs at risk in radiotherapy of rectal cancer patients

**DOI:** 10.1186/s13014-015-0524-3

**Published:** 2015-10-27

**Authors:** Trude C. Frøseth, Trond Strickert, Kjersti S. Solli, Øyvind Salvesen, Gunilla Frykholm, Randi J. Reidunsdatter

**Affiliations:** Department of Oncology, St. Olavs University Hospital, Trondheim, Norway; Department of Cancer Research and Molecular Medicine, Faculty of Medicine, Norwegian University of Science and Technology, Trondheim, Norway; Karolinska University Hospital, Stockholm, Sweden; Department of Health Science, Sør-Trøndelag University College, Trondheim, Norway

## Abstract

**Background:**

The patient positioning in pelvic radiotherapy (RT) should be decided based on both reproducibility and on which position that yields the lowest radiation dose to the organs at risk (OAR), and thereby less side effects to patients. The present randomized study aimed to evaluate the influence of patient positioning on setup reproducibility and dose distribution to OAR in rectal cancer patients.

**Methods:**

Ninety-one patients were randomized into receiving RT in either supine or prone position. The recruitment period was from 2005 to 2008. Position deviations were derived from electronic portal image registrations, and setup errors were defined as deviations between the expected and the actual position of bony landmarks. Setup deviations were expressed into three table shift values (∆x, ∆y, ∆z) from which the deviation vector $\left|\overrightarrow{v}\right|$ were calculated. The estimated lengths of $\left|\overrightarrow{v}\right|$ defined the main outcome and were compared between prone and supine positions using linear mixed model statistics. The mean volume of each 5 Gy increments between 5 and 45 Gy was calculated for the small bowel and the total bowel, and the dose volumes were compared between prone and supine position.

**Results and conclusion:**

Data from 83 patients was evaluable. The mean $\left|\overrightarrow{v}\right|$ was 5.8 mm in supine position and 7.1 mm in prone position (*p* = 0.024), hence the reproducibility was significantly superior in supine position. However, the difference was marginal and may have borderline clinical importance. The irradiated volumes of the small bowel and the total bowel were largest in the supine position for all dose levels, but none of those were significantly different. The patient positioning in RT of rectal cancer patients may therefore be decided based on other factors such as the most comfortable position for the patients.

## Introduction

Colorectal cancer is the second most common type of cancer in Europe, and rectal cancer accounts for a third of colorectal cancer cases [1]. Radiotherapy (RT) in combination with chemotherapy has become the standard adjuvant or neoadjuvant treatment for locally advanced rectal cancer [2, 3]. The efficacy of adjuvant therapies has been demonstrated, in which the addition of chemoradiotherapy (CRT) has specifically reduced the rate of recurrence and improved the survival rate compared to surgery alone [4]. However, this regimen is associated with acute and late small bowel complications that may severely influence the patient’s quality of life [5, 6].

The ultimate goal of RT is to deliver a prescribed dose to a target volume precisely while minimizing dose to the surrounding healthy tissues. To account for the geometrical uncertainties in delivering the target dose the clinical target volume (CTV) is expanded by a total margin (TM) to obtain the planning target volume (PTV). The uncertainties are usually classified as external setup deviations and internal organ movement. Nijkamp et al. quantified the inter-fraction shape variation of the mesorectum of the CTV and found that the differences were small between prone and supine treatments [7]. It follows that the patient setup reproducibility is crucial for the precision of the dose delivery. Reproducibility may be influenced by several factors, such as the physical condition of the patient [8], body mass index (BMI) [9, 10], use of immobilization devices [8], and whether patients are treated in the prone or supine position [11, 12].

Although the prone position has been preferred in order to minimize irradiated bowel volume [13], systematic reproducibility deviations are reported to occur more frequently in this position. The supine position has been associated with more interfractional variation (i.e., random errors) than the prone position [11, 14]. Only one randomized study has investigated patient reproducibility in pelvic RT, and the authors reported that prone position required most corrections [12]. Previous studies addressing the issues of reproducibility in RT of pelvic malignancies have focused mainly on prostate cancer patients [11, 12], and the results may not be readily generalizable to patients with advanced rectal cancer.

The small bowel, the total bowel and the urinary bladder are the most important organs at risk (OAR) in pelvic radiation. The small bowel is the most radiosensitive and dose-limiting normal structure [13, 15]; consequently, diarrhoea is the most common acute side effect of pelvic RT [16–18]. Of patients receiving CRT for rectal cancer, 10 % had to interrupt the scheduled course of RT due to acute gastrointestinal (GI) side effects [15]. Among the more serious late side effects are chronic diarrhoea, perforation, intestinal obstruction, ulceration, fistulas, changed bowel frequency and faecal incontinence [5, 6, 19].

Standard conventional RT to the pelvic region involves irradiation of a considerable volume of small bowel. Several studies have shown associations between dose volumes of the small bowel and acute diarrhoea in patients treated for rectal cancer [16, 17, 20, 21]. Diarrhoea was significantly associated with small bowel volume at all dose levels during preoperative CRT [16, 17, 21], and was strongest associated with volumes receiving doses larger than 15 Gy [16, 20–22]. Furthermore, patients with acute diarrhea (grade 2+ and grade 3+) had significantly larger irradiated small bowel volumes [16, 21].

Given the high level of bowel toxicity in rectal cancer patients and the documented association between toxicity and dose volumes of the small bowel [16, 17, 20, 21], the optimal delivery of RT is important. The most optimal patient positioning should be based on both patient reproducibility and which position yields the lowest possible radiation dose to the OAR and thereby fewer side effects to patients [7]. Therefore, we conducted a prospective randomized clinical study that chiefly aimed to evaluate the effect of patients’ positioning on both setup errors and dose distribution to the OAR.

## Materials and methods

**Fig. 1:**
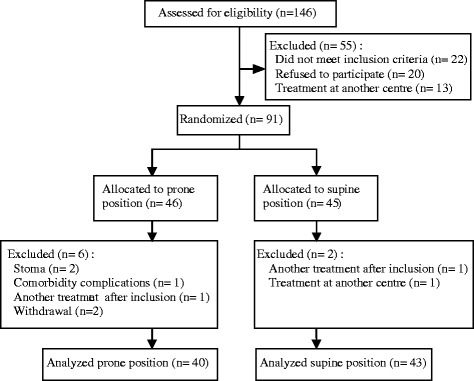
Recruitment flowchart

From November 2005 to December 2008, 146 patients with locally advanced rectal cancer from two hospitals in mid-Norway were invited to participate. Inclusion criteria were the ability to be treated in either the supine or prone position, a WHO performance status of 0 to 2, having both lower limbs intact, and having no hip prosthesis. The study was approved by the regional research ethics committee. After providing written informed consent, participants were randomized into prone or supine positioning using block randomization and stratified according to pre- or postoperative RT. Sample size calculation using two-sample *t-*test estimated that 35 participants in each group were needed to detect a mean difference of 5 mm in the displacement vector in order to provide statistical power of 80 %. The recruitment flowchart is shown in Fig. 1.

### Treatment planning

Computer tomography (CT) scans (3 mm slice thickness) of the whole abdomen and pelvis were obtained in the treatment position. Standard commercial immobilization devices were applied. In the supine position, patients were positioned with a pillow under their heads, knee and ankle support pillows, and their arms resting on their chests. In the prone position, patients were positioned with a pillow under their heads, ankle support pillows, and their arms above their heads. The patients were instructed to empty the bladder one hour before the CT scanning. Gastrografin solution (900 mL) was given orally one hour before scanning to better visualize the bowel for delineation.

A radio-opaque lead pellet anal marker was placed ventrally on the anal verge by an oncologist, and 95 mL intravenous contrast solution (Omnipaque 350 mgI/mL) was given to visualize the vessels. Long lines for alignment were drawn along the three laser lines projected on the patient’s skin. The CT images were imported to the dose planning system (Oncentra MasterPlan v 3.3, Nucletron B.V., Veenendaal, The Netherlands) and the pencil beam algorithm was used for dose calculation. The gross tumour volume (GTV), the CTV and the internal target volume (ITV) were delineated in each CT slice, and the PTV was created according to national guidelines [2]. The internal margin (IM) for creating an ITV was 5 mm in all directions except from the ventral direction, where the margin was 10 mm. The setup margin was 5 mm. CTV included GTV with 10 mm margin (= CTV boost), the mesorectum and the locoregional lymph nodes presacrally, and lymph nodes along the internal iliac vessels up to a cranial border defined by iliaca communis.

**Fig. 2:**
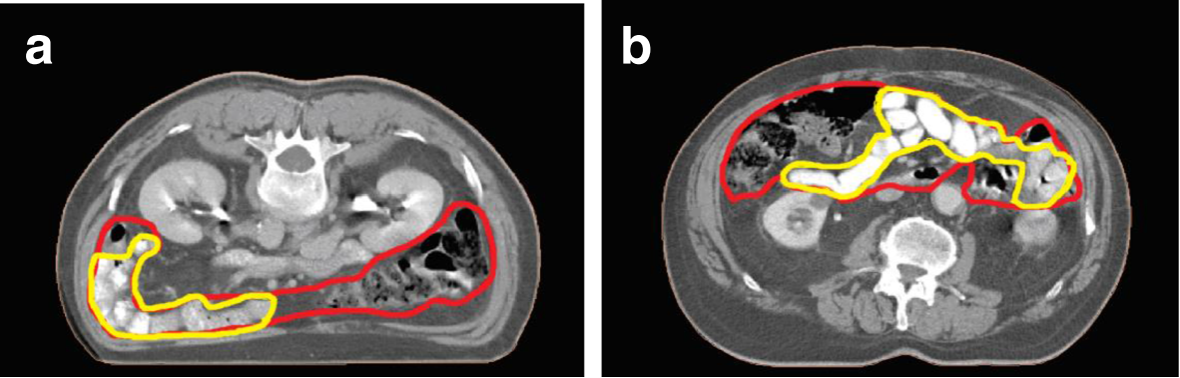
Axial view of a patient showing the small bowel (yellow) and the total bowel (red) in the prone position **a** and the supine position **b**

The small bowel and the total bowel were delineated in every CT slice by contouring the outer extensions of theirs loops and thereby defining the total volume occupied by the organs (Fig. 2). The cranial border of the organs was outlined at the level where the duodenum meets the jejunum. The caudal border was set at the upper level of GTV for preoperative patients and in the upper level of CTV for postoperative patients. The definition of the small bowel did not include the volume of CTV boost because it is a part of the target volume and not an OAR. The urinary bladder was defined by its outer walls.

The notations V5, V10, etc. are used for the volume that receives a dose of 5 Gy or more, 10 Gy or more, etc. The volumes (cm^3^) of small bowel receiving doses in 5 Gy increments from 5 to 45 Gy were calculated using the dose volume histograms (DVH). The calculations were based on the initial pelvic field that received a total dose of 46 Gy because the boost field (up to 54 Gy) did not include the small bowel. Radiation treatment was planned and delivered using a 3-D conformal technique, with either three or four MLC-shaped conform fields delivered by 6 MV or 15 MV photon beams, depending on the proximity of the PTV to the patient’s posterior skin surface. Preoperatively, a mean dose of 46 Gy was given to the ITV in 23 daily fractions, followed by a boost of 4 or 8 Gy to the macroscopic tumour including 10 mm margins. Postoperative RT was delivered by 50 Gy to the ITV in 25 fractions [2].

On the treatment simulator, the isocenter of the planned RT beams was identified and the orientation of the simulator beams were verified against reference digitally reconstructed radiogram (DRR) images. Treatment electronic portal images (EPIs) were obtained of the posterior and lateral treatment fields with *i*View GT EPID (Elekta, Crawley, UK) and were analysed with image-based verification software (VISIR, Nucletron B.V., Veenendaal, the Netherlands). The EPIs were registered with the reference DRRs from the treatment plan. DRR-EPI matching was based upon the identification and outlining of bony landmarks in the pelvic region by two dedicated observers. The linea terminalis and foramen obturatum were delineated in the frontal projection, whereas the ventral part of the sacrum, the acetabulum, and the promontorium were used for lateral beam directions.

Setup errors were defined as deviations between the expected (DRR) and the actual position of the bony landmarks. Setup deviations were translated into table shift values ∆ x, ∆ y, and ∆ z in the three orthogonal directions in a treatment table-oriented coordinate system that corresponded to the lateral, longitudinal, and vertical directions of the table. Setup errors without protocol corrections (intrinsic values) were utilized to cultivate the effect of positioning (removing the protocol effect). Intrinsic values were obtained by adding the foregoing corrections to the measured values. For each measurement, the length of the deviation vector was calculated as:

$$ \left|\overrightarrow{v}\right|=\sqrt{\Delta {x}^2+\Delta {y}^2+\Delta {z}^2} $$

The length of this deviation vector was compared between the prone and supine positions.

### Statistical analysis

The dataset consisted of repeated observations of the setup error vector (i.e., the response variable). The measurements of each patient were made at irregular times (i.e., fractions) according to the interventions dictated by the verification protocol. The logarithmic transform of the vector length was approximately normally distributed and used as the dependent variable in the analysis. A linear mixed model (LMM) was used to examine the effect of treatment positioning on the setup error vector. The log-transformed vector length at fraction *f* for patient *id*, V*f id*, is specified as:

$$ \ln \left({V}_{f, id}\right)={\beta}_{position}+{\alpha}_{id}+{\varepsilon}_{f, id} $$

β is the regression coefficient for the treatment position, and α represents the individual patient effects; α_*id*_ were assumed to be independent and identically normally distributed. The error terms for each patient ε *f* ,*id* were assumed to be multivariate normally distributed with a mean of zero and a covariance matrix defined by:

$$ {\Sigma}_{id}={\left[{\sigma}^2{\rho}^{\left|{f}_i-{f}_j\right|}\right]}_{id} $$

The AR1 autoregressive covariance structure modelled the correlation within each patient. Error terms for different patients were assumed to be independent. The impact of BMI and time (i.e. fraction number) on the setup vector were also tested by including these factors as covariates in the LMM.

Dose volumes and doses to OAR were characterized by their mean values, SD and ranges. For the small bowel and the total bowel, the mean volume of each 5 Gy increment between 5 and 45 Gy was calculated. For the bladder, only V40 and V45 were calculated because of the higher dose tolerance of this organ. Dose volumes were compared between the prone and supine positions using two-sided independent t-tests.

Statistical analyses were carried out using Stata v.12 (StataCorp LP, College Station, TX, USA) and SPSS v.18 (IBM, Armonk, NY, USA).

## Results

Of 91 randomized patients, 83 were included in the study. Of these patients, 40 were treated in the prone position and 43 in the supine position. Patient characteristics are shown in Table 1. All patients received concomitant chemotherapy (5-fluorouracil or Capecitabine), and the great majority (94 %) received RT before surgery. Patient BMI, calculated from their weight at the initial visit, did not differ significantly between the two groups (*p* = 0.29).

**Table 1:** Patient characteristics

	Total	Supine	Prone
No of patients	83	43	40
Preop	78	41	37
Postop	5	2	3
Patients with stoma	11	6	5
Sex			
Male	54	29	25
Female	29	14	15
Age male (years)			
Median	62	62	62
Range	32 – 81	32 – 81	49 – 81
Age female (years)			
Median	63	63	64
Range	52 – 74	51 – 63	51 – 73
BMI (kg/m2)			
Mean (SD)		26.5 (0.2)	26.9 (0.3)
Range		17.2 – 36.3	17.9 – 39.4

### The effect of patient positioning on setup deviations

The mean number of observations per patient was 10.4 (range 6–18) for the prone position and 10.0 (range 7–17) for the supine position. Measurements were made in 43 % of the fractions. Mean deviation values were normally distributed, and the estimates (95 % CI) for the x, y and z directions and the vector are shown in Table 2. The largest errors were observed in the prone position in the vertical direction.

**Table 2:** Mean intrinsic set-up deviations for the lateral, longitudinal and vertical directions

	Mean value (mm)	95 % CI^a^
Lateral (∆ x)		
Supine	0.4	[−0.8, 1.6]
Prone	0.4	[−0.6, 1.4]
Longitudinal (∆ y)		
Supine	0.1	[−0.7, 0.8]
Prone	- 0.3	[−1.3, 0.7]
Vertical (∆ z)		
Supine	0.0	[−1.1, 1.1]
Prone	0.4	[−1.1, 1.8]

^a^ *CI* confidence interval

**Fig. 3:**
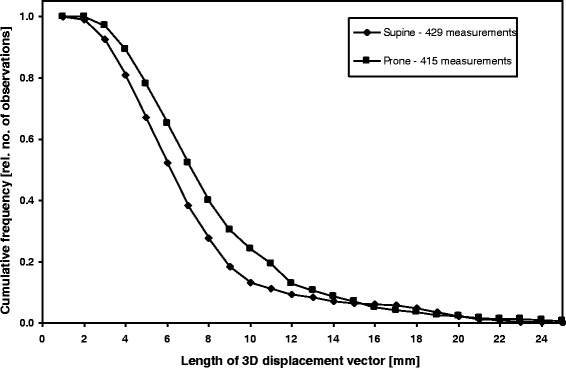
Cumulative frequency distributions of the total displacement vector for supine and prone treatment positions

Cumulative frequency distributions of the deviation vectors illustrate larger deviations in the prone than in the supine position. Frequencies of the greatest deviations (>15 mm) were equal for the two positions (Fig. 3).

The estimated mean vector length (95 % CI) from LMM analysis was 5.8 mm (5.1–6.6 mm) for the supine position and 7.1 mm (6.3–8.1 mm) for the prone position. The mean vector lengths for the two positions were significantly different (*p* = 0.024).

The variance of the log-transformed values of patient effects α_*id*_ as estimated to be 0.372 (*SE* = 0.035), which suggests considerable heterogeneous deviation vector lengths among patients. Variance of the error terms, as a measure of the variation in the setup deviation within patients, was estimated to be 0.473 (*SE* = 0.013). The correlation between error terms for adjacent fractions within patients was estimated to be 0.28 (*SE* = 0.05), which indicates that deviations measured closer in time are more equal. Including time (i.e., fraction number) in the model identified the significance (*p* < 0.001) of this parameter in explaining the variance in the error term. BMI showed a minor and insignificant contribution (*p* = 0.45) explaining the observations. Both time and BMI had negligible effects on the influence on patient positioning on setup errors and were therefore excluded in the final model.

### The effect of patient positioning on dose distributions to organs at risk

Dose distributions for OAR are presented in Table 3. The dose variability was somewhat larger in the prone than in the supine position, but there were no significant differences in the mean doses to the small bowel (p = 0.85), total bowel (*p* = 0.29) or urinary bladder (*p* = 0.74) between the positions.

**Table 3:** Dose volume relations to organs at risk in patients lying in prone or supine position

	Prone				Supine				
Organs at risk	Mean dose ± SD (Gy)	Range (Gy)	Mean defined volume (cm^3^)	Range (cm^3^)	Mean dose ± SD (Gy)	Range (Gy)	Mean defined volume (cm^3^)	Range (cm^3^)	*p*-value*
Small bowel	5.8 ± 5.9	0.4 – 23.2	1451	361 - 2907	6.0 ± 5.2	0.6 - 20.0	1374	793 – 2204	0.85
Total bowel	5.8 ± 4.5	1.1 – 18.9	2977	1718 – 5365	4.8 ± 3.4	0.3 – 13.9	2973	1323 – 5454	0.29
Bladder	33.5 ± 11.7	6.9 – 46.3	211	45 – 647	32.6 ± 10.7	7.1 – 47.3	261	76 – 685	0.74

*Comparisons of mean doses to organs at risk between prone and supine position

The mean defined total volume of the small bowel (Table 3) was somewhat larger in the prone position than in the supine position, while the mean dose volumes at the different dose levels (V5–V45) (Table 4) were somewhat larger in the supine position than in the prone position.

**Table 4:** Dose volume distribution in small bowel and total bowel in prone and supine position

	Small bowel					Total bowel				
	Prone	Supine	Prone	Supine		Prone	Supine	Prone	Supine	
Dose- level	Mean volume ± SD (cm^3^)	Mean volume ± SD (cm^3^)	Proportion irradiated (%)	Proportion irradiated (%)	*p*-value*	Mean volume ± SD (cm^3^)	Mean volume ± SD (cm^3^)	Proportion irradiated (%)	Proportion irradiated (%)	*p*-value*
V5	250 ± 190	305 ± 238	20	23	0.25	486 ± 278	533 ± 326	18	18	0.49
V10	231 ± 189	272 ± 231	19	20	0.39	433 ± 261	460 ± 299	16	16	0.67
V15	159 ± 162	172 ± 198	13	13	0.75	346 ± 254	304 ± 293	13	10	0.49
V20	117 ± 148	124 ± 174	10	9	0.84	277 ± 242	223 ± 269	10	8	0.45
V25	96 ± 135	109 ± 156	8	8	0.70	243 ± 229	192 ± 227	9	6	0.31
V30	81 ± 119	94 ± 131	7	7	0.64	214 ± 206	165 ± 185	8	6	0.26
V35	71 ± 109	84 ± 117	6	6	0.60	192 ± 187	147 ± 163	7	5	0.25
V40	64 ± 102	76 ± 108	6	6	0.60	180 ± 178	135 ± 149	7	5	0.22
V45	50 ± 89	52 ± 74	4	4	0.90	141 ± 149	98 ± 105	6	3	0.14

*Comparisons of mean volumes to small bowel and total bowel between prone and supine position

The dose-volume differences between the prone and supine position increased with decreasing dose and appeared largest at the low-dose volumes (V5 and V10, Table 4). The mean dose-volume differences of the total bowel varied across the dose levels. At V5 and V10, the difference was largest in the supine position, while at V15–V45, the difference appeared largest in the prone position. However, none of these differences was statistically significant. The proportion of irradiated volumes at each dose level was nearly identical in the prone and supine positions.

## Discussion

The present randomized study aimed to establish evidence for the most optimal treatment position with respect to reproducibility and lowest possible dose to OAR in RT of rectal cancer patients. Patients were randomly assigned to either prone or supine positioning. Setup errors were expressed as displacement deviation vectors calculated from table shift values without any protocol corrections in the lateral, longitudinal and vertical directions. The length of this vector was compared between the prone and supine positions. In addition, we explored the dose distributions to OAR for both positions.

Statistically, supine positioning resulted in a significantly smaller mean displacement vector than prone positioning (*p* = 0.024), which indicates the superiority of supine positioning in regard to setup errors. Our results are supported by two studies examining reproducibility in prostate cancer patients [11, 12]. The only truly randomized study, Bayley et al. [12], registered 2-D deviations using daily pretreatment EPI and found that more corrections were needed for patients treated in the prone position. Weber et al. [11] examined 22 prostate patients in a positioning study and reported less 2-D reproducibility for the prone position. Patients with various pelvic malignancies were included in two other studies [10, 14] in which a supine position, overall, turned out to be favorable. In these studies, a belly board was consistently used in the prone position, and there was no randomization regarding the treatment position. Thus, comparisons with previous studies are not straightforward, mainly due to their lack of randomized design [10, 11, 14], small patient numbers [11, 12, 14] and measurements confined to 2-D deviations [11, 12].

Results from LMM analysis revealed substantial variability in the setup deviations both between patients and within individual patients. Variability attributed to the individual patient indicates the considerable influence of random errors on setup deviations. The significant effect of fraction number on set-up deviations confirms the influence of time. Increasing set-up errors by ascending fraction number was also observed by el-Gayed [23]. Deviations from the planning situation may be explained by the patients becoming accustomed to the treatment or that the general condition of each patient deteriorates as treatment proceeds.

Patient BMI had a minor and insignificant contribution to modeling the data variability. This finding was surprising because high BMI values (≥30 kg/m^2^) have been associated with setup deviations in setup error studies [9, 10], which also correspond to the common view in clinical practice that correct positioning is more challenging in obese patients. It might be that the influence of BMI on setup deviations is modified by the patients’ “body firmness.” However, a valid measure of “firmness” has not been introduced in clinical studies.

Setup errors in the prone position were statistically significantly larger than in the supine position. However, the expected difference between prone and supine vector lengths was only 1.3 mm, which probably has marginal clinical significance. We estimated that 35 participants were required in each group in order to detect a vector difference of 5 mm with a statistical power of 80 %. The number of patients actually included in each group was 40 and 43, respectively. The LMM analysis with use of all measurements within patients provided likely a greater statistical power than the two-sample *t*-test used in the initial power calculations based on one mean vector length per participant.

Although the mean doses to the small bowel and total bowel were not significantly different in our data, larger variations were observed in the prone position. Drzymala et al. [13] found that the mean low-dose volumes V5 and V10 were significantly larger in the supine position in a study of 19 patients receiving preoperative CRT for rectal cancer who underwent CT scanning in both the prone and supine positions. Studies utilizing a belly board device have documented significantly lower doses to the small bowel and a lower irradiated volume of small bowel in the prone position than in the supine position [18, 24]. Our insignificant results could be explained by large variations within patients and by the fact that we did not employ a belly board device. However, the benefit of small dose-volume reductions using a belly board has to be considered against reduced reproducibility [8].

The irradiated volumes of the small bowel were largest in the supine position for all dose levels; the differences increased with decreasing dose and appeared largest at the low dose volumes (V5 and V10). However, the proportion of small bowel volume for all dose levels was nearly identical in both positions. Consequently, the likelihood of bowel toxicity is expected to be similar in the two groups. The architecture of the small bowel may be considered as both serial with a dose-dependent risk in a small volume and parallel with a significant correlation between the irradiated volume and the likelihood of acute toxicity [19]. A study of spinal cord in rats demonstrated that high tolerance doses in small regions (shower) decreased significantly when the adjacent tissue was irradiated with a subtolerance dose (bath) [25]. Based on the “shower/bath” theory, not only the high dose volume but also the dose distribution could be determining factors for the risk of toxicity [25]. Although the mean volumes are quite similar, there are relatively large variations in the dose distributions among patients. Hence, it may be worth investigating how the dose-volume distribution correlates with both acute and late toxicity.

Doses to OAR are obviously influenced by the RT technique employed. Volumetric-modulated arc therapy (VMAT) techniques have demonstrated superiority in terms of organs at risk sparing, e.g. small bowel [26]. The advantage of the VMAT technique is largest in postoperative RT when the small bowel often is located in the pelvis and moves less due to inflammatory reactions/fibrosis. The 3D conformal RT is still a routine technique in preoperative treatment. Though the doses to OAR would have been reduced with a VMAT technique, the impact of patient positioning on these doses would probably not been affected.

Our study examined both the small bowel and the total bowel, whereas others have examined the small bowel and the large bowel [20, 22] or only the small bowel [16]. In the present study, we delineated the outer extensions of the bowel loops, and thus defined the total volume occupied by the organ. Some researchers have defined the whole peritoneal cavity [17, 18, 20], whereas others have identified every bowel loop [16, 21]. The latter method results in less defined volume and provides a more true volume of the organ. However, this procedure is obviously more time consuming [18]. The various methods of defining the bowel volumes may explain the different reported associations between defined volumes and acute side effects across studies, and may thus complicate comparisons of the data.

### Limitations and strengths

One limitation of the present study is that the measurements of setup errors were made in only 43 % of the fractions, which may represent a sampling bias. Belly board immobilization could have yielded a smaller bowel dose in the prone position. However, the possible gain in dose reduction has to be weighed against the lack of setup reproducibility by using belly board. The small bowel is a mobile structure and could have changed position during treatment. Cone Beam CT would have provided better visualization of the bowel position over time and would probably unveiled variability in dose delivering to OAR during treatment. However, its clinical efficiency is not fully demonstrated [27]. Excluding CTV from the bowel volume in the postoperative irradiated patients could naturally lead to a false lower bowel dose. However, these patients were few and equally distributed in prone and supine position.

The strengths of the present study are its randomized design and its relatively large number of participants. To our knowledge, we are presenting the first study in rectal cancer patients examining the effect of patient positioning on both reproducibility and dose distributions to OAR.

## Conclusions

Supine positioning is associated with significantly smaller setup deviations than prone positioning in RT of rectal cancer patients. However, the difference is small and may have borderline clinical importance. Doses or dose volumes to the small bowel were not significantly different between the two positions. The optimal treatment position for rectal cancer patients may, therefore, be safely guided by practical considerations and individual factors, such as the most comfortable position for the patient.
